# Regulation of (p)ppGpp and Its Homologs on Environmental Adaptation, Survival, and Pathogenicity of Streptococci

**DOI:** 10.3389/fmicb.2020.01842

**Published:** 2020-09-25

**Authors:** Tengfei Zhang, Jiawen Zhu, Jiajia Xu, Huabin Shao, Rui Zhou

**Affiliations:** ^1^State Key Laboratory of Agricultural Microbiology, College of Veterinary Medicine, Huazhong Agricultural University, Wuhan, China; ^2^Key Laboratory of Prevention and Control Agents for Animal Bacteriosis (Ministry of Agriculture and Rural Affairs), Institute of Animal and Veterinary Science, Hubei Academy of Agricultural Sciences, Wuhan, China; ^3^Institute of Animal Sciences, Chengdu Academy of Agricultural and Forestry Sciences, Chengdu, China; ^4^International Research Center for Animal Disease (Ministry of Science & Technology of China), Wuhan, China; ^5^Cooperative Innovation Center of Sustainable Pig Production, Wuhan, China

**Keywords:** streptococci, (p)ppGpp synthetase, physiology, pathogenicity, regulation

## Abstract

Most streptococci are commensals, pathogens, or opportunistic pathogens for humans and animals. Therefore, it is important for streptococci to adapt to the various challenging environments of the host during the processes of infection or colonization, as well as to *in vitro* conditions for transmission. Stringent response (SR) is a special class of adaptive response induced by the signal molecules (p)ppGpp, which regulate several physiological aspects, such as long-term persistence, virulence, biofilm formation, and quorum sensing in bacteria. To understand the roles of SR in streptococci, the current mini-review gives a general overview on: (1) (p)ppGpp synthetases in the genus of *Streptococcus*, (2) the effects of (p)ppGpp on the physiological phenotypes, persistence, and pathogenicity of streptococci, (3) the transcriptional regulation induced by (p)ppGpp in streptococci, and (4) the link between (p)ppGpp and another nutrient regulatory protein CodY in streptococci.

## Introduction

Bacteria in the genus *Streptococcus* are Gram-positive cocci-shaped organisms organized in chains, and include *Streptococcus pyogenes*, *Streptococcus pneumoniae*, *Streptococcus mutans*, *Streptococcus suis*, *Streptococcus equisimilis*, *Streptococcus agalactiae*, and others. Most *Streptococcus* bacteria are human or animal commensals, pathogens, or opportunistic pathogens (Sader et al., 2006; Palmieri et al., 2011). During the processes of infection or colonization, bacteria suffer various challenges in stress and nutrient insufficiency in the host environment (Cotter and Hill, 2003; Kaspar et al., 2016). Typically, the blood plasma of the host provides abundant nutrients; in contrast, the interstitial tissue fluid contains much lower concentrations of free amino acids, glucose, free inorganic phosphates, and metal ions, which are required for the growth and persistence of streptococci (Zhang et al., 2012). In addition, streptococci may encounter more severe nutrient shortages when initially contacting the epidermal tissues or when persisting at a high cell density in the nidi of infection or inside host cells (Steiner and Malke, 2000). The stresses from the host also include high temperatures, an acidic environment, reactive oxygen species (ROS) stimulation, and other factors (Zeng et al., 2011; Abranches et al., 2018; Korir et al., 2018). Therefore, responses to these environmental cues are important factors with respect to colonization and disease progression.

Bacteria have evolved efficient stress response mechanisms to adapt to challenging environments. Among these responses, a special class of adaptive response induced by (p)ppGpp is called “stringent response (SR)” (Potrykus and Cashel, 2008). A wide array of physiological aspects, such as long-term persistence, virulence, biofilm formation, and quorum sensing, have been reported to be affected by (p)ppGpp (Taylor et al., 2002; Dahl et al., 2003; Kazmierczak et al., 2009; Geiger et al., 2010). Species of the *Streptococcus* genus are associated with public health and veterinary medicine concerns. An understanding of the SR of streptococci will facilitate the development of tools and means of controlling these pathogens. In this review, we provide information on (p)ppGpp synthetases and (p)ppGpp-mediated adaptation responses on the physiological phenotypes, persistence, and pathogenesis, as well as the global regulation in streptococci.

## The (p)ppGpp Synthetases in Streptococci

Nearly 50 years ago, (p)ppGpp was discovered in *Escherichia coli* as two “magic spots.” The (p)ppGpp is synthesized by RelA/SpoT homologous proteins (RSH) through transferring a pyrophosphate moiety from adenosine triphosphate (ATP) to guanosine diphosphate (GDP) or guanosine triphosphate (GTP; Cashel, 1974, 1975; Avarbock et al., 2000). Two RSH enzymes, RelA, and SpoT, are involved in (p)ppGpp synthesis in *E. coli*. The RelA has (p)ppGpp synthetic activity and is recognized to respond to amino acid starvation, and the SpoT has both synthetic and hydrolytic activities, and senses many other environmental stressors such as starvation of carbon, iron, phosphate, and fatty acids (Seyfzadeh et al., 1993; Vinella et al., 2005; Potrykus and Cashel, 2008).

**Figure 1:**
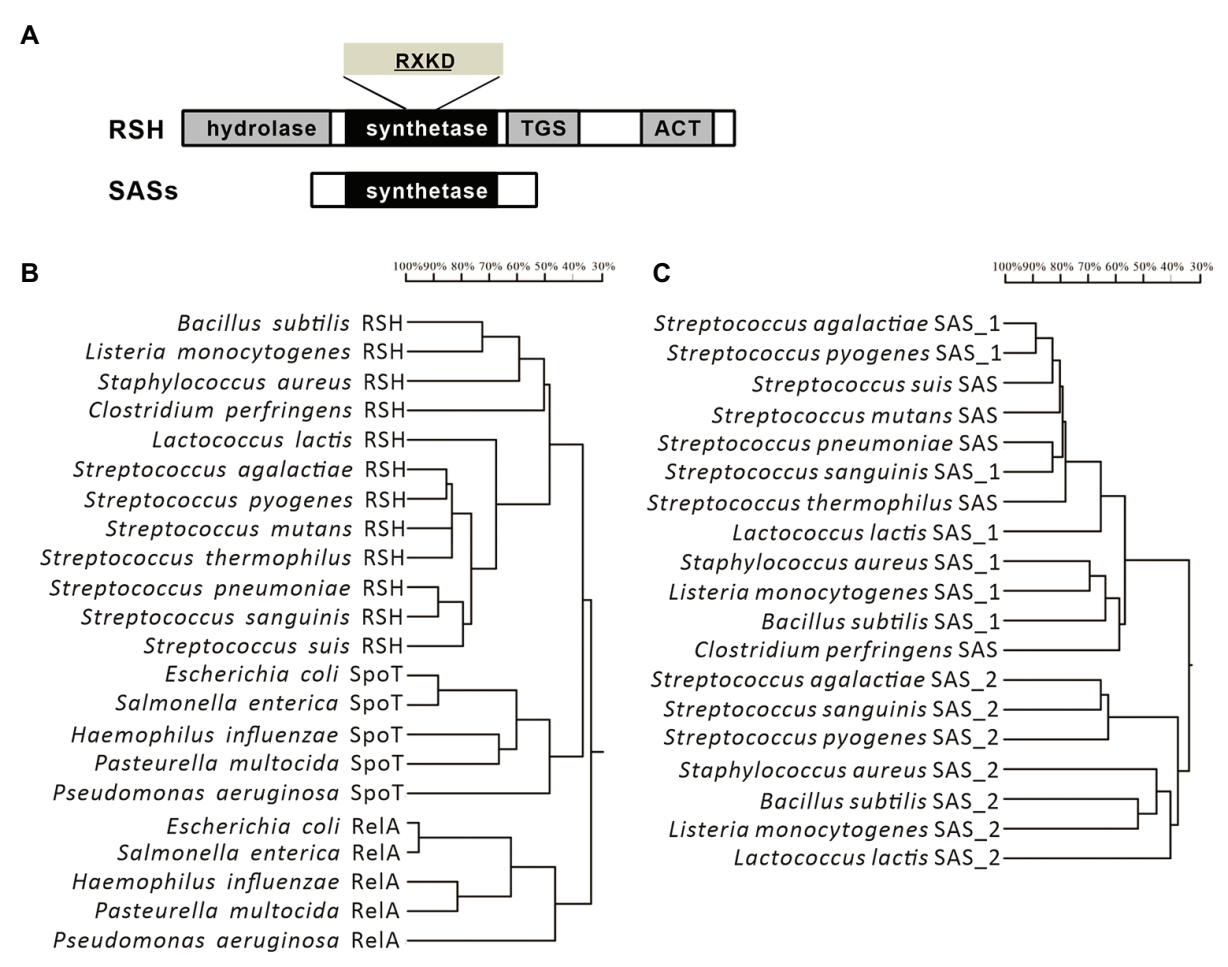
Domain structures and phylogenetic trees of the long RelA/SpoT homologous proteins (RSH)-type synthetases and SASs from streptococci. **(A)** Domain structures of the long RSH-type synthetases and SASs from streptococci. **(B,C)** Phylogenetic trees of the long RSH-type synthetases and SASs from streptococci and other species of bacteria.

In streptococci, the RSH protein is firstly characterized in *S. equisimilis* (Mechold et al., 1996), and subsequently identified in *Streptococcus rattus*, *S. pyogenes*, *S. mutans*, *S. pneumoniae*, *S. agalactiae*, and *S. suis* (Mechold et al., 1996; Whitehead et al., 1998; Lemos et al., 2007; Nascimento et al., 2008; Kazmierczak et al., 2009; Zhang et al., 2016). Different from *E. coli*, which encodes two long RSH-type synthetases (Steinchen and Bange, 2016), streptococci only contain a single long RSH-type synthetase, usually named Rel, such as Rel*_Smu_* in *S. mutans* (Lemos et al., 2007), Rel*_Spn_* in *S. pneumoniae* (Kazmierczak et al., 2009), Rel*_Spy_* in *S. pyogenes*, Rel*_Ss_* in *S. suis* (Whitehead et al., 1998; Zhang et al., 2016), and Rel*_Seq_* in *S. equisimilis* (Mechold et al., 2002). The long RSH-type synthetase in streptococci contains four main domains (Figure 1A). The N-terminal region is the catalytic part acting as both hydrolysis and synthesis domains of (p)ppGpp, and the C-terminus is recognized as the regulatory region including TGS and ACT domains. According to the amino acid sequences, the long RSH-type synthetase in streptococci contains a RXKD motif, which is a conserved basic motif found in the bi-functional RSH proteins, and shows higher similarity with SpoT rather than the RelA of *E. coli* (Figure 1B; Sajish et al., 2007; Zhang et al., 2016). According to the function, the long RSH-type synthetase in streptococci has a strong (p)ppGpp hydrolytic activity and a weaker (p)ppGpp synthetic activity, also much like the SpoT in *E. coli* (Lemos et al., 2007; Sajish et al., 2007). Similar to the RelA in *E. coli*, (p)ppGpp synthetic activity of RSH in Gram-positive bacteria can be activated by interacting with idling ribosomes during amino acid starvation (Avarbock et al., 2000). However, the mechanisms of stringent responses induced by various stressors are different, but not well-characterized (Potrykus and Cashel, 2008).

Compared to *E. coli*, although one long RSH-type synthetase (like RelA in *E. coli*) which mainly has the (p)ppGpp synthetic activity is absent, some small/short proteins with only (p)ppGpp synthetic activity (Small Alarmone Synthetases, SASs) have been identified in *Firmicutes*, including most species of streptococci (Figures 1A,C; Lemos et al., 2007; Nanamiya et al., 2008). The numbers of SASs in streptococci vary in different species. For example, two SASs, RelP, and RelQ, are encoded in the genome of *S. mutans* (Lemos et al., 2007), and among them, RelP is the primary enzyme to synthesize (p)ppGpp during exponential growth and co-transcribed with a two-component signal transduction system (TCS) RelRS (Lemos et al., 2007), while *relQ* is in a four-gene operon, which is essential for persistence and pathogenesis of *S. mutans* (Kim et al., 2012). In contrast, we only found one SAS, RelQ, encoded in the genome of *S. suis*, and we verified that it can synthesize (p)ppGpp under amino acid starvation, but it is non-functional under glucose starvation (Zhang et al., 2016). A previous study has reported that the SAS of *Enterococcus faecalis* synthesizes a ppGpp molecule with more efficient function than pppGpp (Gaca et al., 2015), but this differentiation has not been studied in streptococci yet.

## The Effects of (p)ppGpp and Its Homologs on Streptococcal Physiology

**Figure 2:**
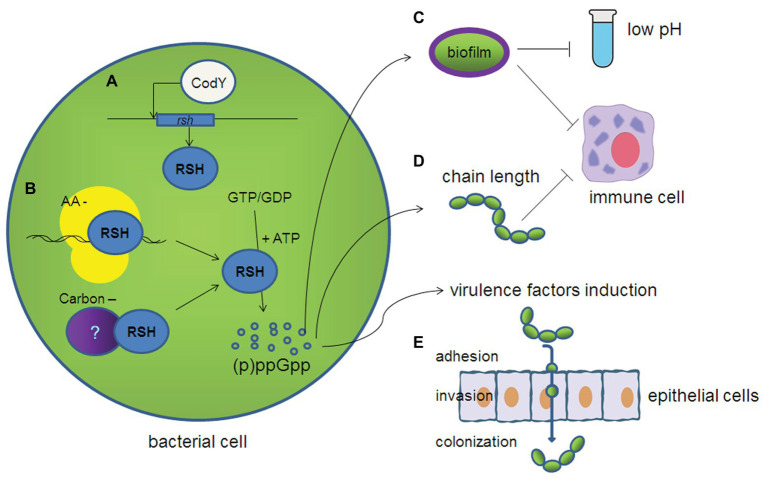
Diagram depicting the various roles in streptococci affected by (p)ppGpp. **(A)** CodY can positively regulate the expression of *rel_Ss_* in *Streptococcus suis*. **(B)** (p)ppGpp synthesis can be triggered by the interaction of ribosomes and (p)ppGpp synthetase during amino acid starvation, but the trigger mechanisms under carbon starvation and other stress conditions are still unknown in streptococci. **(C)** (p)ppGpp contributes to the biofilm formation and confers the resistance to acid stress and immunocyte killing. **(D)** (p)ppGpp is essential for maintaining its chain length, which make it less likely to be uptaken by immune cells. **(E)** (p)ppGpp contributes to adhesion and invasion to the epithelial cells by regulating the expression of various virulence factors.

(p)ppGpp, which acts as a signaling molecule to cause a stringent response, plays an important role in its environmental adaptation. This response is involved in several physiological phenotypes in streptococci, including growth and cell morphology (Figure 2). Usually, although the growth of *rsh* mutants is a little slower compared with wild-type strains, RSH is not essential under nutrient replete conditions in most of the streptococci (Lemos et al., 2007; Kazmierczak et al., 2009; Zhu et al., 2016). In contrast, (p)ppGpp is accumulated in wild-type cells under amino acid or glucose starvation, leading to the arrest of growth or slow growth, while RSH inactivated strains show a higher growth rate than wild-type strains at the beginning of starvation, but then evolve more quickly into the stationary phase (Kazmierczak et al., 2009; Zhu et al., 2016). These results suggest an energy saving process for long-term survival under starvation stress through a (p)ppGpp dependent regulation. In particular, the *rel_Spn_* mutant of *S. pneumoniae* requires copper and manganese for growth, due to the lack of metabolic adjustment caused by (p)ppGpp (Kazmierczak et al., 2009). The *rel_Ss_* is also identified as an upregulated gene under iron-restricted conditions using selective capture of transcribed sequences (SCOTS) in *S. suis* in our lab, which suggests the role of Rel*_Ss_*/(p)ppGpp in iron regulation (Li et al., 2009). The chain arrangement of cells is the typical characteristic of streptococci, and we found that the chain length of *S. suis* becomes longer in the Δ*rel_Ss_*Δ*relQ* mutant (Zhu et al., 2016). The chain length of Δ*cpsA* is observed to be longer than the wild-type strain in *S. agalactiae* (Hanson et al., 2012). The increased chain length of Δ*rel_Ss_*Δ*relQ* mutant in *S. suis* may be attributed to the regulation of (p)ppGpp in the capsular biosynthesis cluster (*cps*) cluster, according to our transcriptome result (Zhang et al., 2016).

## The Effects of (p)ppGpp and Its Homologs on Persistence and Pathogenicity

Most species in streptococci are pathogens for animals and/or humans. Resistance to the unfavorable environment as well as innate and acquired immunity is the key point of persistence for pathogens. As a cariogenic bacterium, adaptation to acid stress is important for the persistence of *S. mutans* in the oral cavity, and biofilm formation is a major factor in this process (Wang et al., 2017). Biofilm formation of the Δ*rel_Smu_* mutant is reduced in *S. mutans*, and it is interesting that, although there is no difference in the acid sensitivities between the wild-type and Δ*rel_Smu_* strains grown in planktonic cultures, when cells are grown in biofilms, the Δ*rel_Smu_* mutants become more acid resistant than the wild-type strain, and it is directly related to increased glycolytic capacities (Lemos et al., 2004). We have confirmed that the survival of Δ*rel_Ss_*Δ*relQ* mutant of *S. suis* is also reduced in whole blood and it is more sensitive to phagocytosis of THP-1 monocytic leukemia cells (Zhu et al., 2016). One of the reasons may be that the increasing chain-length of the Δ*rel_Ss_*Δ*relQ* mutant make it more sensitive to complement deposition, and then to uptake by immune cells (Dalia and Weiser, 2011). Variation of RSH in *S. pneumoniae* is found to be associated with phenotypic differences based on a genomic diversity analysis, and researchers further confirmed that RSH confers higher resistance to neutrophil-killing (Li et al., 2015). In *S. agalactiae*, Rel*_Sag_* is also essential to its survival in blood (Hooven et al., 2018). These results suggest the important roles of Rel*_Sag_*/(p)ppGpp in the resistance to immune killing by the host.

As described above, (p)ppGpp/RSH confers a high resistance ability to streptococci, which aids in host persistence, and further facilitates the expression of virulence factors, as well as infection and pathogenesis. The *rel_Spn_* of *S. pneumoniae* is proven to be a major virulence factor in a murine pneumonia/bacteremia model of infection (Hava and Camilli, 2002), and researchers further confirmed that Rel*_Spn_* confers a higher competitiveness in mouse colonization (Li et al., 2015). It is noteworthy that the Δ*rel_Spn_* mutant is not only attenuated, but also the progression of infection is dramatically altered. The first sign of disease is changed from lung to groin or in the abdomen and caused by the Δ*rel_Spn_* mutant. The change in infection progression may be due to the lack of stringent response, which plays an important role in the adaptation to the host’s internal environment, including metabolic changes and the availability of metal ions (Kazmierczak et al., 2009). The intercellular communication between the ComX inducing peptide (XIP) and (p)ppGpp is also identified in *S. mutans*, and this cross-communication is involved in the virulence-related phenotypes, including modulated competence signaling and development (Kaspar et al., 2016). In our tests in *S. suis*, disruption of *rel_Ss_* and *relQ* leads to decreased adhesive and invasive abilities to Hep-2 cells, and mouse infection experiments show that the Δ*rel_Ss_*Δ*relQ* mutant is attenuated and becomes easier to be cleaned up by the host (Zhu et al., 2016). In *S. agalactiae*, Rel*_Sag_* is essential to its survival in blood, and the *rel_Sag_* knockout strains demonstrated a decreased expression of beta-hemolysin, which is implicated in invasion of this pathogen (Hooven et al., 2018).

## Transcriptional Regulation *via* (p)ppGpp During Stress Response

In *E. coli*, accumulation of (p)ppGpp in concert with DksA, results in alterations in gene expression, owing to changes in RNAP activity during stress conditions (Potrykus and Cashel, 2008). In contrast to *E. coli*, the effect of (p)ppGpp on rRNA transcription is independent of DksA homologs in Gram-positive bacteria including streptococci (Kazmierczak et al., 2009). The (p)ppGpp synthesis seems to decrease rRNA transcription indirectly through depletion of the GTP pool, which is the initiating nucleotide in rRNA transcripts (Krasny and Gourse, 2004; Kazmierczak et al., 2009). The global regulation of RSH/(p)ppGpp has been investigated through the transcriptome analysis under amino acid and/or glucose starvation in some species of streptococci. The common and typical transcriptome feature is adjusting bio-macromolecular synthesis and transport in response to nutrient availability (Nascimento et al., 2008; Zhang et al., 2016). For example, during glucose starvation, lots of genes associated with protein synthesis, DNA replication and cell division were repressed, while carbohydrate transporters were upregulated under the control of Rel*_Ss_*/(p)ppGpp in *S. suis* (Zhang et al., 2016). In *S. pneumoniae*, the majority of *rel_Spn_*-dependent genes were associated with translation and ribosome structure, amino acid metabolism and transport, and DNA replication and repair (Kazmierczak et al., 2009). These regulations match the classical stringent response in *E. coli* and other bacteria (Potrykus and Cashel, 2008).

At the same time, the transcriptome of each *streptococcus* also shows their unique characteristics, which are associated with their adaptation and/or pathogenesis (Figure 2). In *S. pneumoniae*, Rel*_Spn_* and (p)ppGpp amounts play wide-ranging homeostatic roles in pneumococcal physiology, and the operon encoding the major exotoxin pneumolysin is also under the regulation of (p)ppGpp/Rel*_Spn_* (Kazmierczak et al., 2009). In *S. agalactiae*, the transcription levels of the arginine deiminase (*arcA*) pathway are decreased during stringent response, while arginine availability modulates the expression of cytotoxicity, which is important for virulence (Hooven et al., 2018). During glucose starvation, besides the classic stringent response including inhibition of growth and related bio-macromolecular synthesis, the extended adaptive response includes inhibited glycolysis, and carbon catabolite repression (CCR)-mediated carbohydrate dependent metabolic switches in *S. suis* in our tests (Zhang et al., 2016). In addition, the expression of some virulence-related genes of *S. suis*, such as *cps*, glyceraldehyde-3-phosphate dehydrogenase (*gapdh*), fibronectin-binding protein (*fbps*), enolase (*eno*), *arcA*, VicR response regulator, type IV-like secretion system component (*virD*4), superoxide dismutase (*sod*), muramidase-released protein (*mrp*), extracellular protein factor (*epf*), and suilysin (*sly*) are downregulated in the Δ*rel_Ss_*Δ*relQ* mutant (Zhu et al., 2016). In *S. mutans*, Rel*_Smu_* also plays a major role in the regulation of phenotypic traits, which are required for persistence and virulence expression of this oral pathogen (Nascimento et al., 2008). In addition, a 5-fold downregulation of *luxS* gene in the *rel_Smu_* mutants suggests a link between the AI-2 quorum sensing and stringent response in *S. mutans* (Lemos et al., 2004).

Although (p)ppGpp is critical for the adaptation of starvation, a specific subset of genes involved in pathogenesis and metabolism were both modulated in the RSH mutants as well as in wild-type streptococci, suggesting the important roles of RSH-independent responses during stress conditions. For example, the regulation of a TCS *covRS*, exotoxin B regulator *ropB*, oligopeptide (*opp*), and dipeptide (*dpp*) permease systems, and *pepB* involved in the intracellular processing of oligopeptides, are RSH-independent during amino acid starvation in *S. pyogenes* (Steiner and Malke, 2000). In *S. mutans*, the expression of 50 genes involved in functions including energy metabolism and TCSs and others, is commonly affected in wild-type and Δ*rel_Smu_* mutant strains after Mupirocin treatment (Nascimento et al., 2008).

## Linkage Between (p)ppGpp and Cody in Streptococci

Amino acid starvation not only induces a stringent response but also CodY mediated regulation. Many common phenotypes can be regulated by both (p)ppGpp and CodY, suggesting their potential coordinated regulation (Geiger et al., 2010). GTP is not only the substrate of (p)ppGpp synthetases, but also acts as a ligand to enhance the affinity of CodY and its target DNA in lots of bacteria, like *Listeria monocytogenes*. Therefore, RelA-dependent (p)ppGpp accumulation reduces the GTP pool, and further leads to a reduction of DNA binding ability of CodY in cells (Geiger and Wolz, 2014). In the Δ*relA* mutant of *L. monocytogenes*, the increase in the GTP pool can reduce the expression of the CodY regulon and virulence, but deletion of *codY* from the Δ*relA* strain can restore its virulence (Bennett et al., 2007). In *Bacillus subtilis*, the lower GTP level imposed by stringent response also results in the de-repression of CodY target genes (Handke et al., 2008).

According to prior studies of *S. pyogenes* and *S. mutans*, GTP is not a co-factor for CodY. This may suggest that the linkage between (p)ppGpp and CodY is particularly different in streptococci (Malke et al., 2006; Lemos et al., 2008). We further confirmed this point in *S. suis* (Zhu et al., 2019). Of note, CodY can interact with the *rel_Ss_* promoter in a GTP-independent manner and act as a transcriptional activator to positively regulate the *rel_Ss_* expression in *S. suis* (Figure 2; Zhu et al., 2019). Real-time RT-PCR showed that the deletion of the *codY* gene in the Δ*rel_Ss_* strain further reduced the expression of virulence factors of *S. suis* compared to Δ*rel_Ss_*, and the lethality and colonization of the Δ*rel_Ss_*Δ*relQ*Δ*codY* strain in mice were significantly reduced as well. This may suggest a new interplay between the (p)ppGpp synthetase and CodY in *S. suis* (Zhu et al., 2019).

## Perspectives

As the (p)ppGpp-mediated stringent response is critical in the adaptation, survival, and pathogenesis of streptococci, the (p)ppGpp related pathways are potential targets to control pathogens and their infections. To understand the mechanism of that, it is important to understand how the (p)ppGpp synthetase control the levels of (p)ppGpp pools in cells. This has been partly revealed in the model microorganism *E. coli*. The SpoT in *E. coli* interacts with the acyl carrier protein (ACP), the central cofactor of fatty acid synthesis, which is involved in sensing the signals of fatty acid starvation and carbon starvation (Battesti and Bouveret, 2006). Another study found that Rsd directly interacts with SpoT and stimulates its (p)ppGpp hydrolase activity (Lee et al., 2018). However, the stringent response in streptococci is sometimes different from that in *E. coli*, and largely unknown. For example, the interaction between RSH and ACP did not occur in *S. pneumoniae* (Battesti and Bouveret, 2009). Therefore, the sensing mechanisms of the stringent response during fatty acid and carbon starvation are still unknown in streptococci. Discovering the interactions between (p)ppGpp synthetases and stress receptors are key research points for future studies of streptococci. Another strategy for discovering antibacterial agents based on stringent response is the use of a (p)ppGpp analogue, such as Relacin, which can reduce (p)ppGpp production (Wexselblatt et al., 2012, 2013). The inhibition function of Relacin has been verified in *Bacillus* (Wexselblatt et al., 2012); whether it is functional in streptococci is still unknown.

## Author Contributions

TZ and RZ conceived the study and wrote the manuscript. JZ and JX provided the figures. HS revised the manuscript. All authors contributed to the article and approved the submitted version.

### Conflict of Interest

The authors declare that the research was conducted in the absence of any commercial or financial relationships that could be construed as a potential conflict of interest.
